# Accurate Representation of Protein-Ligand Structural Diversity in the Protein Data Bank (PDB)

**DOI:** 10.3390/ijms21062243

**Published:** 2020-03-24

**Authors:** Nicolas K. Shinada, Peter Schmidtke, Alexandre G. de Brevern

**Affiliations:** 1 SBX Corp., Tokyo-to, Shinagawa-ku, Tokyo 141-0022, Japan; shinada@sbx-corp.com; 2 Discngine SAS, 75012 Paris, France; peter.schmidtke@discngine.com; 3 INSERM, UMR_S 1134, DSIMB, Univ Paris, INTS, Laboratoire d’Excellence GR-Ex, 75015 Paris, France

**Keywords:** protein-ligand complexes, dataset, clustering, structural alignment, refinement

## Abstract

The number of available protein structures in the Protein Data Bank (PDB) has considerably increased in recent years. Thanks to the growth of structures and complexes, numerous large-scale studies have been done in various research areas, e.g., protein–protein, protein–DNA, or in drug discovery. While protein redundancy was only simply managed using simple protein sequence identity threshold, the similarity of protein-ligand complexes should also be considered from a structural perspective. Hence, the protein-ligand duplicates in the PDB are widely known, but were never quantitatively assessed, as they are quite complex to analyze and compare. Here, we present a specific clustering of protein-ligand structures to avoid bias found in different studies. The methodology is based on binding site superposition, and a combination of weighted Root Mean Square Deviation (RMSD) assessment and hierarchical clustering. Repeated structures of proteins of interest are highlighted and only representative conformations were conserved for a non-biased view of protein distribution. Three types of cases are described based on the number of distinct conformations identified for each complex. Defining these categories decreases by 3.84-fold the number of complexes, and offers more refined results compared to a protein sequence-based method. Widely distinct conformations were analyzed using normalized B-factors. Furthermore, a non-redundant dataset was generated for future molecular interactions analysis or virtual screening studies.

## 1. Introduction

Protein structures are the support of essential biological functions. They are highly dynamic macromolecules and adopt an ensemble of conformations during their lifetime. Multiple resolution techniques have been elaborated to access their three-dimensional structures. X-ray crystallography and Nuclear Magnetic Resonance spectroscopy (NMR) are the most common and efficient resolution methods. The obtained structures are stored and freely available for the scientific community in the Protein Data Bank (PDB) [1], a widely used public database since the 1970s. A significant increase in structure deposition throughout its existence is observed, e.g., going from 54,000 protein structures in 2008 to 160,000 in 2020. PDB does not exclusively contain protein structures, ligands are also displayed in PDB structures, resulting in a larger number of protein-ligand complexes. They are widely used in structure-based drug discovery [2]. Structures of ligand complexes are used for drug design purpose, e.g., they can be used to train scoring functions of protein-ligand interactions [3]. They are also critical in the understanding of the underlying principles of intermolecular interactions, e.g., the recent analyses of halogen interactions between proteins and ligands [4]. These structures are also often utilized to benchmark novel methods in the realm of molecular modeling.

Nevertheless, a major difficulty to perform a proper benchmark for a specific method using resources, such as the PDB, is to ensure an unbiased protein dataset, i.e., specific non-redundant datasets must be produced. Multiple methodologies exist to evaluate and generate such non-redundant protein datasets using underlying amino acid sequence information, e.g., PDBSelect [5] or PISCES [6]. Heuristics have also been proposed to be quick and usable for large datasets, e.g., BLASTCLUST [7] or CD-HIT [8]. Only a very limited number tried to take into account the protein structure, e.g., PAPIA [9]. Today, tools available on the PDB website allow non-redundant dataset retrieval using sequence similarity measures alone. As protein structures are, in a certain extent, subjectively created models, their recurrence can improve the confidence for a structure. Even so, these repetitions, or redundancy, can induce bias. It is widely acknowledged within the PDB by the scientific community, yet ill-considered. The only studies related to this subject are focused on conformational ensembles, such as NMR structures, corresponding to 8.5% of the PDB [10,11], which are, by definition, highly similar models.

While most of the previous methods focus only on protein sequences, proteins bound to DNA, to RNA, to small molecules, or to amino acids containing post-translational modifications (PTM) [12] are more difficult to analyze due to their diversity. For instance, in structures of protein–DNA complexes, proteins can have easily reach thousands of amino acids while a DNA structure of more than 15 bp is rare [13]. The situation is similar to protein-ligand complexes and directly affects their analyses.

Today, a few tools exist to gather proper protein-ligand complexes datasets. The Binding Mother of All Databases (MOAD) [14] includes 25,769 high-quality (resolution better than 2.5 Å and biologically relevant ligands) protein-ligand complexes taken from the September 2017 PDB. They address the question of redundancy by looking at the protein sequence and using molecular fingerprints coupled with Tanimoto coefficient regarding the ligands [15]. PDBBind [16] provides yearly releases and contains currently 17,900 biomolecular complexes in the 2017 version. They proposed a limited number of proteins defining a ‘core set’ to try to handle the question of redundancy curated manually [17]. The scPDB [18], an annotated database of binding sites in the PDB, contains 4782 proteins and 6326 ligands in its 2017 release. In its original publication [19], absence of redundancy is mentioned in their dataset without provided metrics. While these databases offer refined protein structures, none of them explore and assesses the structural diversity of their complexes.

Previous work by Wallach and Lilien in 2009 [20] already focused on this particular issue. To improve the quality of binding models extracted from PDB complexes, a non-redundant dataset was generated, considering sequence similarity for the protein part (BLASTp) and small molecule fingerprint similarity metrics. However, they do not consider cases where identical ligands bind to different binding sites on the same protein. Furthermore, no structural assessment was performed in their study. The last update of the dataset was performed in 2013. Drwal and coworkers have recently published a study on 2911 complexes from the PDB including 1079 fragments and 1832 small molecules highlighting fragment binding mode conservation in 74% of the dataset [21]. Small element substitutions on fragment have little to no impact to the fragment-binding mode and interaction patterns appear to be maintained. 

Here, we propose a first quantitative evaluation of the structural redundancy observed in PDB focusing on protein-ligand complexes. Basic statistics on overrepresented proteins and molecules are derived. A specific clustering is performed to define the accurate number of unique complexes resulting in the generation of a refined dataset for molecular interaction studies or virtual screening protocols. Finally, we discuss and illustrate some of the surprising findings.

## 2. Results

### 2.1. Initial Dataset

#### 2.1.1. Statistics

**Figure 1:**
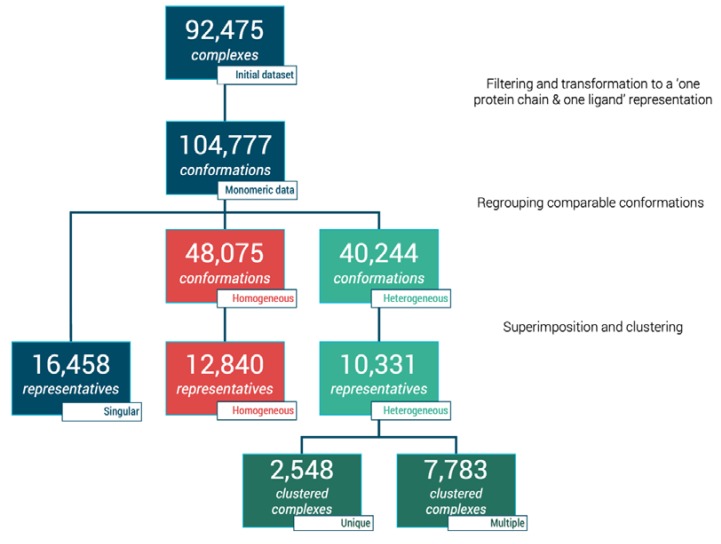
Flowchart of dataset clustering and characterization of protein–ligand complexes.

The initial database query retrieved 110,735 interacting complexes from 3decision™ software. Multiple filtering steps such as ligand size, single bond ratio, protein chain size, and number of residues in contact with ligand (as described in Materials and Methods) were performed. This phase contributed to a reduction of our initial dataset to 92,475 protein-ligand complexes (see Figure 1). These protein-ligand sets are spanned across 39,411 PDB entries. At this stage, one ligand can be bound to more than one protein chain; thus, each case of multimeric complex was then separated culminating in 104,777 individual protein-ligand conformations, i.e., monomeric data (see Figure 1). These units were then analyzed to define different sub-datasets.

#### 2.1.2. Diversity

**Figure 2:**
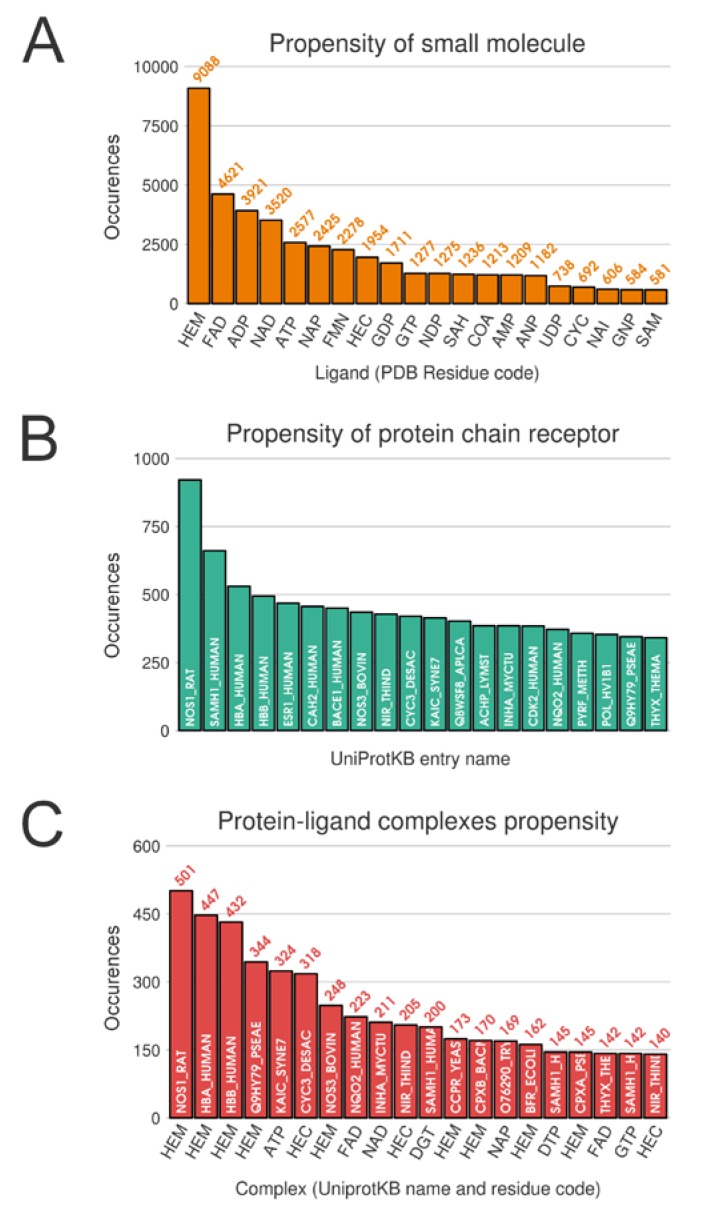
Top 20 distributions in monomeric dataset. (**A**) Distribution of ligands. (**B**) Distribution of protein chain (with UniProt IDs). (**C**) Distribution of protein-ligand complexes (with UniProt IDs and residue code).

Unsurprisingly, from the multimeric complex dataset, the most represented ligand in the PDB is the heme (PDB residue code Protoporphyrin IX Containing FE (HEM)) with 9088 occurrences, accounting for a representation of 7.9%. Expected popular ligands are present among the 10 most frequent molecules in the PDB, such as nucleobase derivatives, e.g., Adenosine-5’-Triphosphate (ATP), Nicotinamide-Adenine-Dinucleotide (NAD), Flavin Mononucleotide (FMN), S-Adenosyl-L-Homocysteine (SAH) (see Figure 2A). Interestingly, using SMILES identity, a large number of 17,135 unique small molecules are found. Consequently, this unbalanced ligand distribution is reflected among our protein representation where nucleotide coenzymes receptors and heme receptors are also frequently observed (Figure 2B). The strong occurrences of these specific ligands and receptors culminate logically in a large presence of their corresponding complexes. Heme bound to nitric oxide synthase, with respectively 9,088 and 921 occurrences, are represented by 501 distinct conformations (see Figure 2C).

### 2.2. Singular Protein-Ligand Complexes

The simplest type of complexes to analyze are the singular complexes as they were defined as such when no other identical ligand or identical proteins were found for these complexes, namely the *singular* dataset. Moreover, 15.7% of our dataset (16,458 out of 104,777 monomeric complexes) fit this description (see Figure 1).

Distribution of ligands among the singular dataset reveals nucleobase-like molecule remains the top representation with a combined 739 occurrences for Phosphoaminophosphonic Acid-Adenylate Ester (ANP), AMP, Adenosine-5’-Diphosphate (ADP), and ATP. Heme is slightly underrepresented compared to its overall distribution in our initial dataset with 174 cases for HEM residue code and 152 instances for Heme C (HEC) residue code. Moreover, 84.7% of these small molecules have only one representation in this unique dataset.

**Figure 3:**
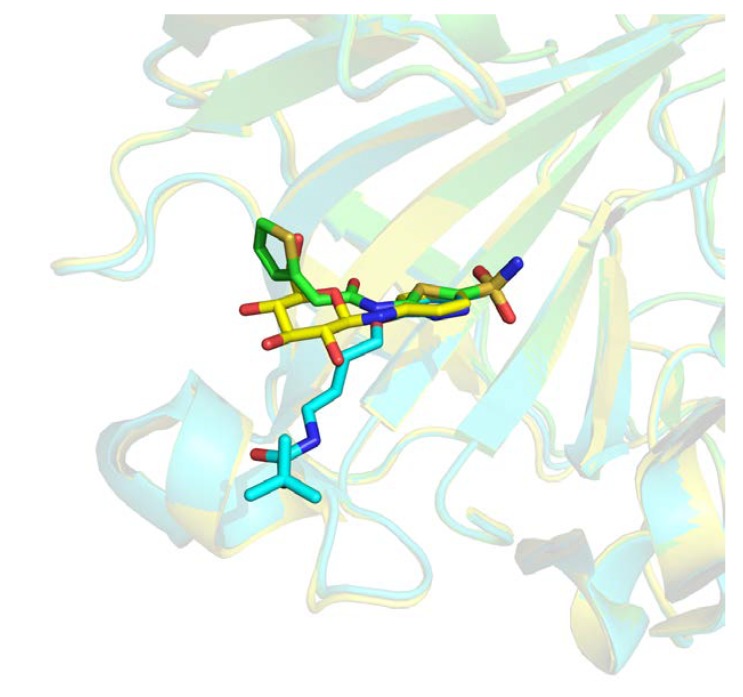
Three-dimensional (3D) representation of three different ligands bound to Carbonic Anhydrase 2 receptor (Protein Data Bank (PDB) IDs 4iwz in green, 2rfc in blue, 2hl4 in yellow).

Those 16,458 complexes represent 5239 distinct protein chains, 2232 only present once in this subset. The remaining 3007 protein chains feature, on average, 4.73 distinct ligands with a distribution largely unbalanced. As an example, two distinct binding sites of the carbonic anhydrase enzyme are interacting with 259 distinct ligands. One of these binding sites is illustrated in Figure 3, where three different ligands are shown interacting with the same pocket. Similar redundancy is observed for prothrombin, β-secretase 1, and cyclin-dependent kinase 2 with, respectively, 204, 199, and 195 occurrences.

**Figure A1:**
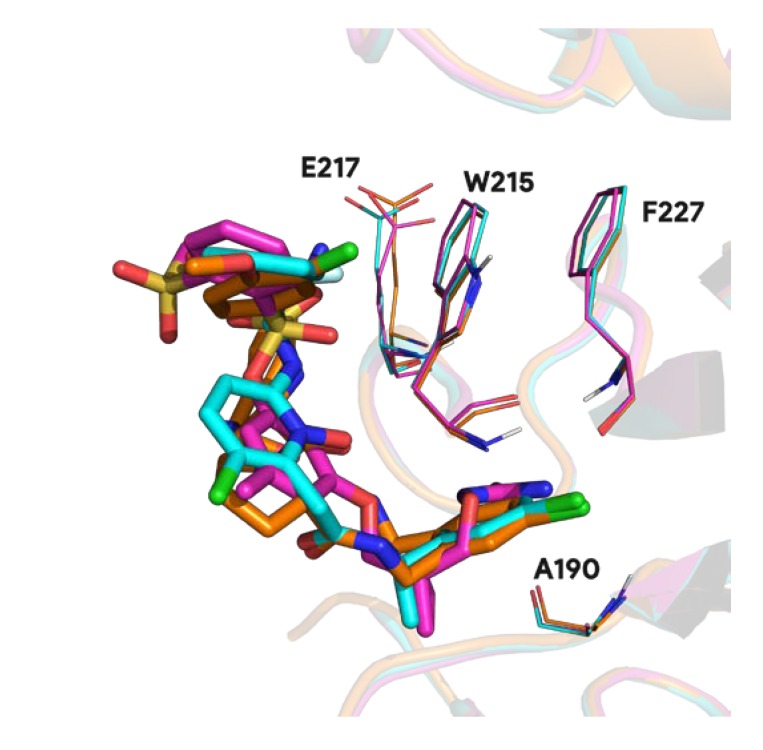
3D visualization of conserved residues (lines) in the prothrombin ligand (sticks) binding process (PDB ids 1t4u in pink, 1z71 in blue and 3u9a in orange).

These singular complexes shown here highlight general tendencies towards specific protein targets in biological research field. Recurrent proteins bound with distinct ligands underlines important binding residues and conserved molecular fragment used in ligand optimization. For instance, 95.1% of prothrombin proteins found in the PDB involve residue W215 and 94.1% A190 in the binding mechanism. Other less frequent residues involve E217 in 55.0% of complexes and F227 in 24.5% of complexes (see Figure A1).

### 2.3. Protein-Ligand Complexes Groups

The remaining units represent 88,319 conformations, i.e., 84.2% of our dataset. Conformations with identical ligands, protein chains, and similar binding sites were grouped and compared to each other within the group. 

**Figure 4:**
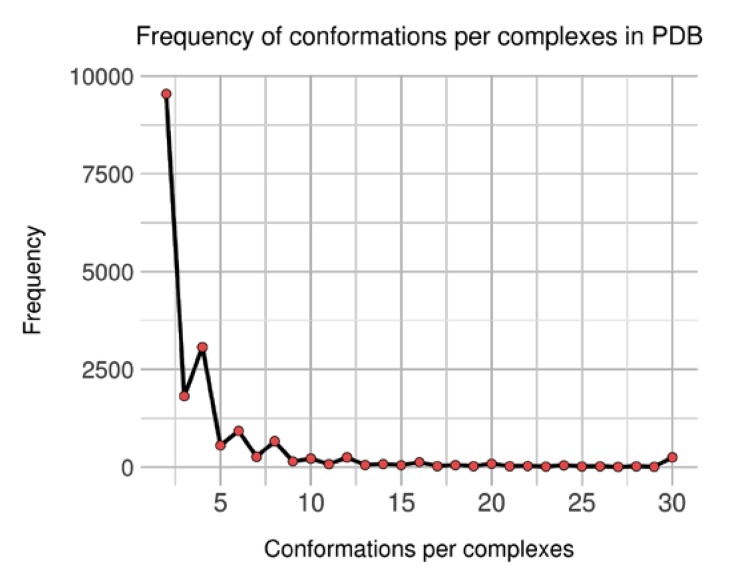
Distribution of observed conformations per complexes in non-singular dataset.

At first, an initial number of 18,478 groups (of complexes) were generated containing between 2 and 501 conformations. As each group features at least two units, distribution of conformations count per complex was analyzed (see Figure 4). It shows an unbalanced representation, where complexes with few conformations are predominant: the 9542 of groups (51.6%) featuring two conformations represent 21.6% of the 88,319 conformations. Still, complexes with more than 30 conformations tally for 17.8% conformations and only 1.4% of the groups; the biggest group is again for brain nitric oxide synthase, with 501 occurrences. 

Then, a structural alignment and quantification was performed between each unit within one group on common binding residues, e.g., $\frac{n\left(n-1\right)}{2}$ comparisons per group with *n* being number of conformations. Over 1,130,000 superimpositions and weighted fragment root mean square deviation (wRMSD_f_) computations were performed. Groups were then split between homogeneous binding modes, i.e., identical conformations, and heterogeneous binding poses, i.e., sharing some similarity.

#### 2.3.1. Homogeneous Complexes

Homogeneous complexes are defined as groups for which each conformation is identical to each other, i.e., conserved binding modes. Each comparison among one group results with a wRMSD_f_ value below 1.0 Å and no distance between aligned fragments was greater than 1.5 Å.

Moreover, 12,840 groups were considered as homogeneous in our dataset, equivalent to 48,075 units (45.9% of our monomeric dataset, see Figure 1). As those complexes display identical binding modes, a 3.75-fold reduction can be processed when considering only one representative per group, i.e., 12,840 unique representatives.

Multiple group sizes are represented across these complexes. A large number of redundant conformations for one complex is expected in NMR structures. However, the largest group is composed of 173 units across 158 unique PDB X-ray entries. One representative of this heme bound to mitochondrial cytochrome C peroxidase complex is available through PDB entry 1aen. 

**Figure 5:**
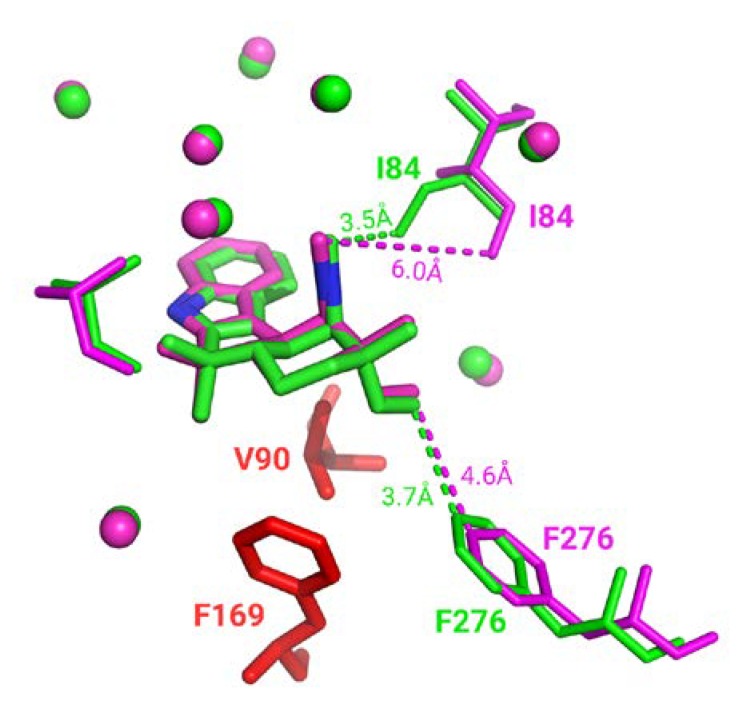
Three-dimensional representation of WelO5 protein in complex with 6CU ligand stick representation (PDB IDs 5iqv green and 5iqu magenta). Residues in PDB entry 5iqu that are unresolved in PDB entry 5iqv are colored in red. Closest distances observed are shown in dashes. Superposed residues are displayed in spherical representation.

Almost every superimposition made in the homogeneous subset is associated with a good binding residues alignment. However, some interesting cases, 160 complexes (1.6%), displayed a significant number of distinct binding residues; thus, not selected for superimposition. Specific visual inspection of those cases indicates: (i) due to protein inner flexibility, these binding residues are identified in only one conformation as the detection depends on a distance threshold (e.g., highlighted by distance in Figure 5) and (ii) other residues can be unresolved in one of the structures (corresponding to residues colored in red in Figure 5). It must be noted that since ligand-binding modes remain identical in homogeneous groups, missing residues and ambiguous residue detection do not impact neither binding site superposition nor assessment.

This descriptor describes the similarity in pocket shape. Moreover, 97.1% of our homogeneous comparisons display a pocket RMSD less than 0.5Å, i.e., structurally conserved pocket. Overall, structure superposition result in identical pocket shape with an average pocket RMSD of 0.18Å with a standard deviation of 0.13 Å.

**Figure 6:**
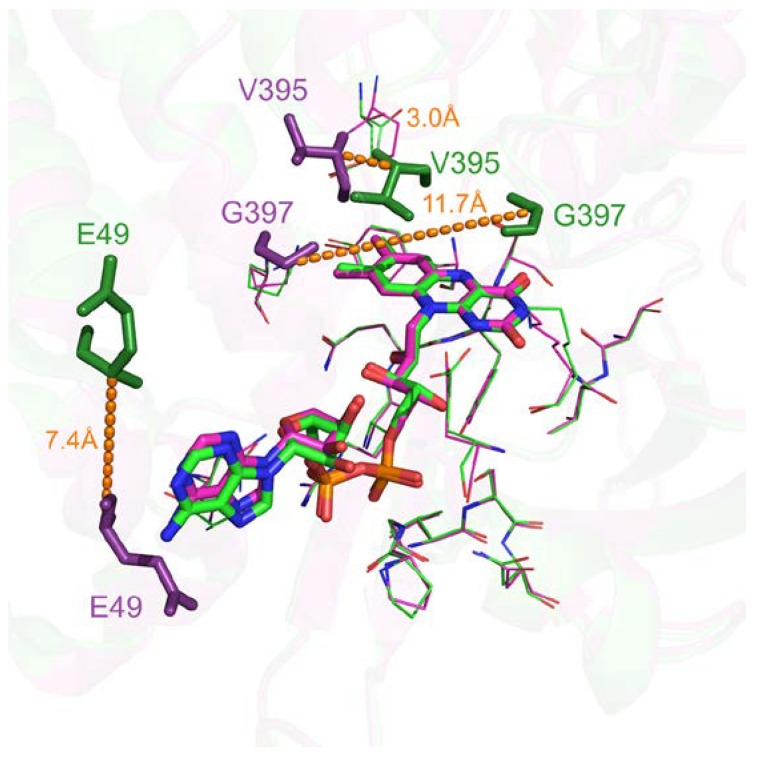
Three-dimensional representation of superposed Flavin-Adenine Dinucleotide (FAD) binding sites of flavin adenine dinucleotide receptor. Structurally conserved residues are displayed in ‘lines’ representation. Flexible residues are displayed in ‘stick’ representation and in darker colors with their corresponding deviation highlighted by dashes (PDB IDs 1cqx in green and 3ozv in purple).

Only 615 comparisons out of 233,417 (0.26%) have a pocket RMSD greater than 1.0 Å. These specific cases are largely caused by flexible secondary structure, such as loop highlighted in Figure 6. Visual inspection of flavin adenine dinucleotide receptor with Flavin-Adenine Dinucleotide (FAD) ligands shows a significant number of binding residues that are conserved. However, residues such as G397 belong to a flexible loop leading to a 11.7 Å distance between the two Cαs after alignment (highlighted by orange dashes on Figure 6). E49 and V395 also displayed significant differences with 7.4 Å and 3.0 Å shifting. Interestingly, these deviations can highlight either potential multiple binding roles due to their proximity with the ligand in either cases or space filling characteristics.

#### 2.3.2. Heterogeneous Complexes

Finally, the remaining 5638 complexes corresponding to 40,244 conformations (38.4% of our dataset), present at least one comparison where either wRMSD_f_ is greater than 1.0 Å or one distance between compared fragments above 1.5 Å. To avoid unnecessary clustering, if every comparison within one group was greater than 2.0 Å, complexes were automatically considered as distinct. Clustering was performed in situations where both high and low wRMSD_f_ values were observed to extract the representatives poses.

**Figure 7:**
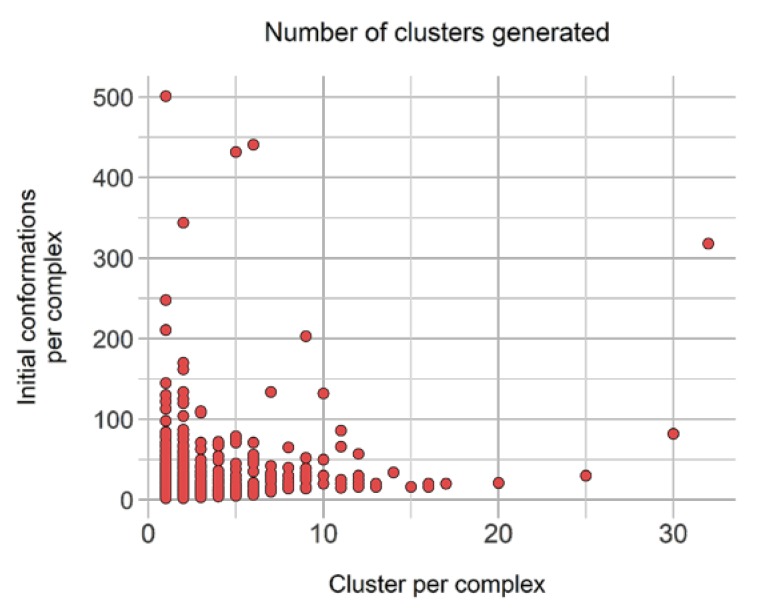
Distribution of number of generated clusters relative to the original binding poses count to be clustered in group B. Propensity of cluster sizes.

Filtering and clustering resulted 10,331 distinct binding modes across those 40,244 conformations, corresponding to an interesting 3.89-fold reduction. Moreover, three subgroups were distinguished from these: (i) 2548 complexes (17,602 conformations) with only one cluster, i.e., one representative, (ii) 2360 unique conformations from complexes with only distinct conformations (wRMSD_f_ > 2.0 Å), and (iii) 5423 representative conformations originated from complexes with scattered clusters size. The latter accounts originally for 2006 complexes represented by 20,283 conformations, with a high number of complexes represented by less than five similar representatives (Figure 7). 

Figure 7 underlines the number of representative units generated from clustering relative to the number of initial conformations available. Interestingly, despite having initially 22 complexes with more than 100 initial distinct conformations, none leads to more than 35 conformations, and only four have more than 20 distinct conformations. 

The most redundant complex in our dataset, 501 distinct structures of heme bound to brain nitric oxide synthase, was clustered into one representative binding modes, with an average wRMSD_f_ of 0.37 Å (standard-deviation 0.18).

**Figure 8:**
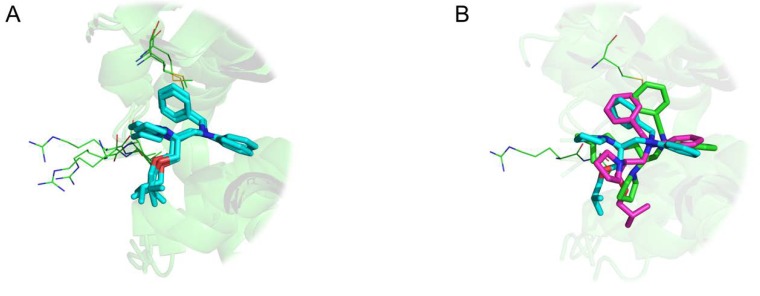
Three-dimensional representation of Cardiac Troponin with Bepridil (PDB ID 1lxf), ligand represented in ‘stick’ representation, binding residues in ‘lines’ representation: (**A**) Cluster of three identical ligand conformations (average weighted fragment root mean square deviation (wRMSD_f_) 0.67 Å), (**B**) three distinct conformations extracted from same NMR structural model (average wRMSD_f_ 2.90 Å).

Intriguing results arise especially in the study of NMR structures. For instance, Figure 8 highlights 12 distinct binding modes identified for xylose isomerase protein (PDB id 1xlf) among 30 protein-ligand conformations. The biggest cluster being composed of six similar units. A cluster of three conformations in Figure 8A displays excellent superposition with an average wRMSD_f_ of 0.73Å, while alignments on other representatives display significant structural deviation (three examples highlighted in Figure 8B). 

Finally, structure assessment on small clusters was performed using B-factors. Clusters featuring three or more identical conformations were considered as structurally stable and therefore devoid of bias in atom positioning during resolution. For the remaining clusters, with three or less conformations, B-factors were extracted and normalized, corresponding to 7672 X-ray structures with a resolution greater than 1.5 Å.

**Figure 9:**
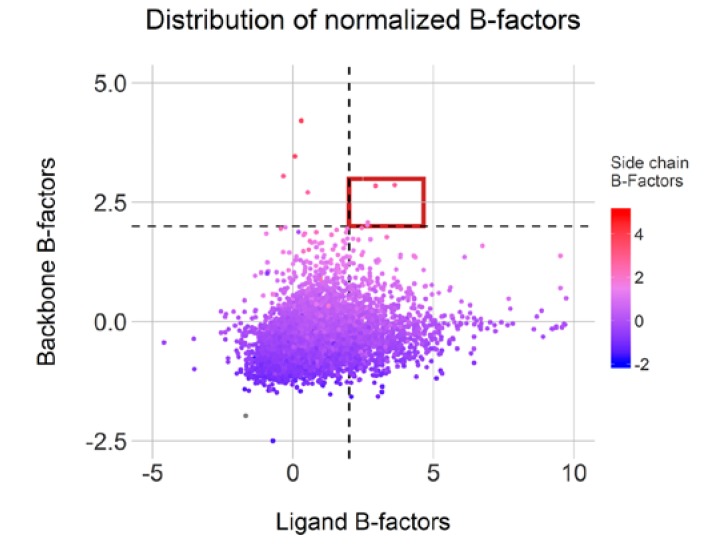
Normalized B-factor distributions for complexes with distinct conformations (high Root Mean Square Deviation (RMSD) values) and complexes with less than three clustered conformations. Cases with high flexibility in ligand and pocket are highlighted in red.

Moreover, 1199 conformations displayed a ligand normalized B-factor value above 2.0Å^2^, an arbitrary threshold but described as clearly flexible by Bornot et al. [22]. The average normalized B-factor values calculated on both backbone and side-chain residues indicate mostly rigid or intermediate environment for these ligands. Surprisingly, only four cases (highlighted in red in Figure 9) display both mobile ligand and binding sites and only 77 cases featured flexible side-chains (above 2.0 Å^2^). This observation illustrates an overall binding site rigidity in opposition to ligand flexibility. Overall, 1211 conformations from our initial 7642 representatives can be categorized as cautionary due to high flexibility in either the ligand or protein counterpart. Of course, the positioning of some of these ligands could be attributed to low resolutions or poor fitting of the ligands in the electron density map.

### 2.4. Non-Redundant Dataset Generation

This study focusing on binding modes diversity results in the generation of a non-redundant dataset of protein-ligand complexes based on those refined binding modes with no systematic bias. The criteria to select the representative for each cluster is an aggregate ranking between structure resolution, the maximum number of contacting residues and the averaged wRMSD_f_ for each conformation. Following the previous described step (singular dataset and representative of the two categories of non-singular datasets), the proposed non-redundant dataset of 39,629 complexes. Hence, this supervised approach leads to a pertinent and significant 2.64-fold reduction over the initial dataset. The final list (see Appendix A) contains PDB IDs, ligand residue code, and number as defined in the PDB, and their corresponding conformation in case of NMR structures or alternate conformations.

A critical comparison must be done with the most classical approach to define a non-redundant dataset, i.e., a simple sequence identity threshold without consideration of ligand. It would have produced a dataset of 9997 complexes, i.e., a considerable loss in regard to the final proposed dataset. Associating ligand similarity to the process, 30,873 distinct complexes would have been generated, a 22.1% decrease compared to our final non-redundant dataset. This discrepancy comes from multiple circumstances, such as (i) considering multiple binding sites per protein chain with the same ligand generating specific instances (ii) differentiating conformations through structural comparison of distinct binding modes.

Our final dataset includes 31,846 complexes with only one binding mode observed in the PDB and 4366, characterized by multiple representative units. Moreover, 95.0% of those recurring complexes have less than five representative conformations. Similarly to sequence-based approach, our non-redundant dataset still retains 9997 distinct protein chains. Carbonic anhydrase 2, β-secretase 1, and cyclin-dependent kinase 2 proteins are among the recurrent complexes identified with 332, 297 and 272 occurrences respectively. These values are mostly due to the diversity of ligands crystallized with these proteins of high interest.

**Figure 10:**
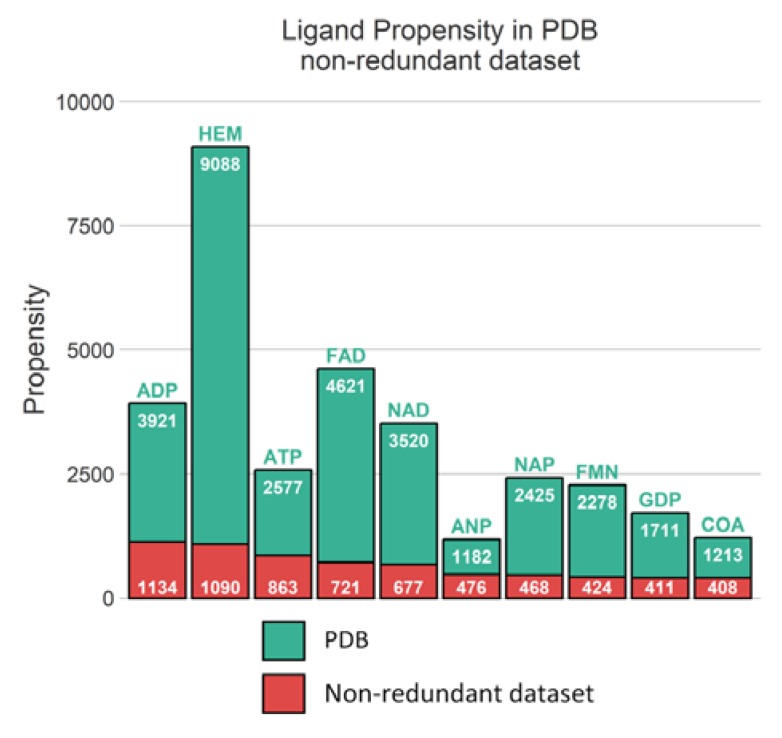
Distribution of 10 most frequent ligands observed in PDB and in non-redundant dataset.

To note, 16,771 distinct ligands are identified, with heme still being one of the most recurrent ligands. However, compared to the 9088 instances of heme observed in our initial dataset, clustering has reduced heme representation by a factor 8.4 (see Figure 10). Similarly, flavin adenine ligand (FAD) occurrences have been reduced by 6.4-fold indicating a significant redundancy for the most frequent small molecules. Hence, it should be noted that this difference of 22% in terms of occurrence reflects the fact that the lists are very different. In the list based only on the sequences, the cases noted as redundant are taken as a single entry. Likewise, related proteins are automatically eliminated even if they can be involved in very different interactions.

## 3. Discussion

Throughout this study, we’ve highlighted the diversity and conservation of ligand binding poses in complex with identical protein. The number of distinct binding modes in the PDB can be reduced to 39,629 complexes, a significant decrease compared to the initial dataset. 

While protein-ligand datasets were generated in the past such as scPDB [19], they only focus on specific criteria such as resolution, drug-like ligand, and binding residue. Generation of similar dataset using PICSES webserver can result in more than 130,000 protein chains, but mainly do not take into account the question of the ligand as it is only using protein sequence [6].

Our analysis features both structural analysis and supervised dataset generation that can be used to various degrees. Results and non-redundant dataset, available in supporting information, can be used for multiple purposes such as highlighting conserved and mobile residues in the binding mechanism for specific protein (see Figure 6). Ligand diversity for one specific binding site (see Figure 3) can also be easily explored using our results. Using the distinct representative conformations of one complex (displayed in Figure 8) to refine ensemble-docking results is another way our results can be exploited in the future. Our approach takes into account the possibility that a specific ligand interacts with different sites of the same protein. This opportunity had been rarely taken into account, but could be of great help in the search for catalytic, binding, or regulatory allosteric exosite [23,24].

The multiple levels of redundancies observed across PDB relative to protein-ligand context have also been underlined. Specific protein chains are largely overrepresented and were analyzed thoroughly in our dataset. Hence, the 501 conformations of the most recurrent complex, heme bound to brain nitric oxide synthase, are all structurally identical from a binding perspective. Consequently, the 9088 occurrences of heme complexes can be represented by 1090 unique binding modes.

Furthermore, our methodology is not biased by small discrepancies in the protein chain. Missing residues or insertion of a chimeric peptide still lead to similar binding modes that are well retrieved in our study. Structures available in poor resolutions can be validated by the co-occurrence of other redundant structures with high structural similarity for instance. We can also notice the specific interest of clusters of distinct ligand conformations. Indeed, they can reflect attractive dynamics and specifics of the binding affinity. Using different computational approaches such as scoring docking functions and molecular dynamics, they would be stimulating cases to apprehend their different binding affinities.

**Figure 11:**
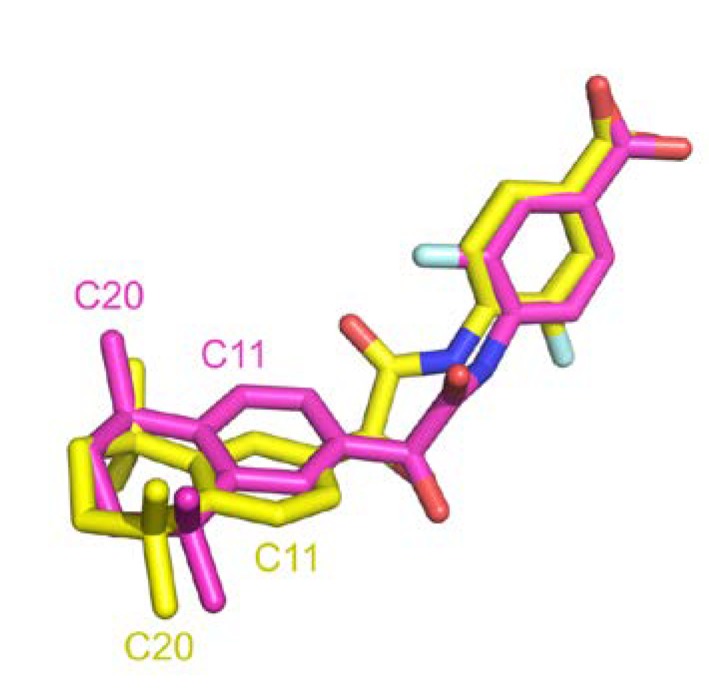
Atom label inverted in two PDB entries (PDB IDs 4lbd in magenta and 1exx in yellow).

A comparison between structural similarity metric can also be discussed. RMSD computed with atoms is generally used in structural comparison studies. This approach requires matching predefined atom labels to compute deviation distance. We were surprised to found high RMSD for many entries that were clearly identical. Indeed, sometimes atom labels were inverted across different entries leading to high distance over perfectly superposed fragment. Figure 11 illustrates such instance where after superposition, two ligands are aligned but atom annotation in PDB are completely inverted in rings, e.g., C11 or F1 atoms, resulting in an atomic RMSD of 2.9 Å compared to a wRMSD_f_ value of 0.8 Å. Pearson correlation coefficient computed across these two metrics in our dataset resulted in a correlation factor of 0.64, indicating a moderate correlation. Using wRMSD_f_ in our case avoid this type of bias induced by annotations. 

Nonetheless, fragment RMSD has its own limitations, aromatic rings plane rotation for instance aren’t well characterized. Two perpendicular aromatic rings superimposed on their center of mass will not be quantitatively different despite the potential change in interaction geometry and, consequently, nature.

**Figure 12:**
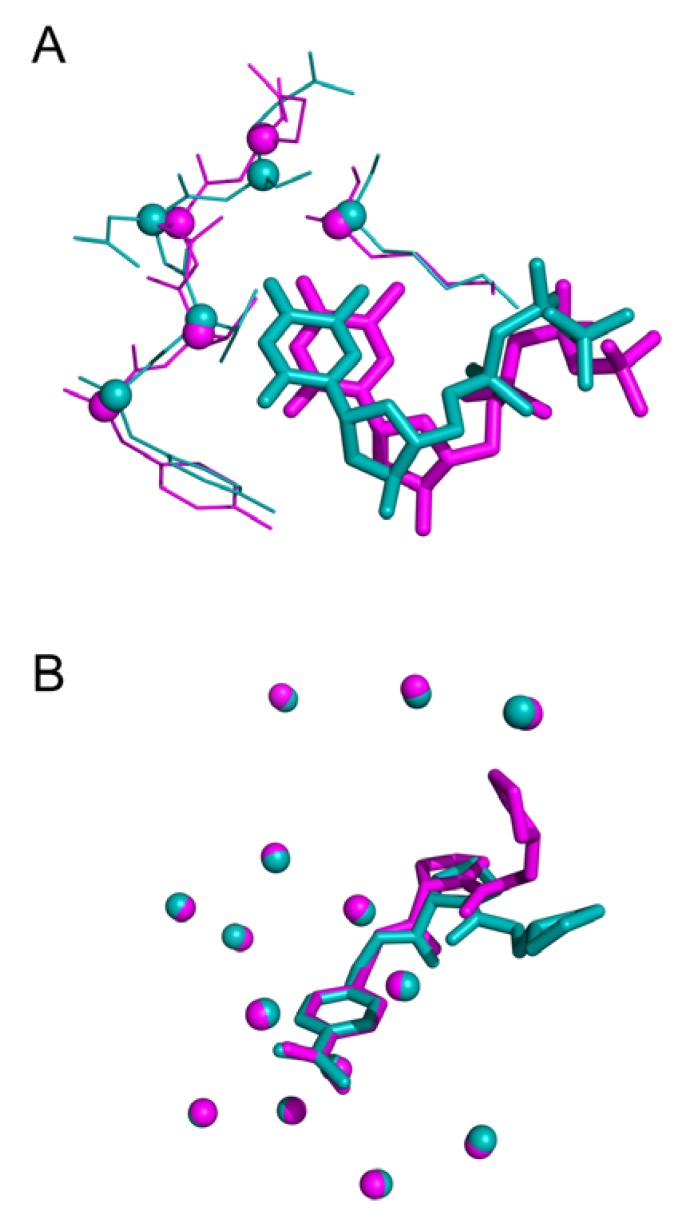
Three-dimensional representation of (**A**) effector TTP deviation bound to Ribonucleotide (PDB IDs 3hnd in blue and 3hnf in magenta, wRMSD_f_ 2.08 Å), **(B)** fragment shift in trypsin S3 pocket (3ljj in blue 2zft in magenta, wRMSD_f_ 1.97 Å).

Threshold used to define structural similarity can also be discussed. Indeed, evaluation of docking approach often uses a RMSD (atom-based) value less than 2.0 Å to consider a ligand pose similar to the native state. However, RMSD represent an average-like measure of similarity. Therefore, a value of 2.0 Å can highlight various cases: (i) a shift of the entire molecule as illustrated in Figure 12A (ii) a structurally conserved ligand region in the binding site and a significant deviation of some fragments in Figure 12B.

## 4. Materials and Methods

The 128,843 protein structures were downloaded from the Protein Data Bank website; they were obtained by X-ray crystallography, NMR and cryogenic electron microscopy (cryo-EM) methods. They were processed and analyzed using our knowledge-based database Discngine 3decision™. Regarding NMR ensembles, each model was processed separately as a distinct protein-ligand conformation. Structures were annotated using multiple sources, such as UniProt [25], ChEMBL [26], PFAM [27], and Prosite [28]. Each structure was assigned to one (or multiple, e.g., alternative splicing events or chimeric protein) UniProt reference sequence(s) using the structure residue sequence and UniProt reference sequence. 

Those annotations allowed a precise description of the specificity of one particular protein chain, macromolecules and ligands, mainly defined as heteroatoms, across multiple structure entries. No condition filter was applied regarding the ligand type, natural or designed ligand. This decision, purely subjective, was done to offer the broadest possible view of the data available to the scientific community. Similarly, the 3decision software automatically detected crystal contacts observed at the ligand level, and ligands whose positioning was suspicious were not considered for the study. For our current study, complexes were selected by narrowing to a set of specific ligands. Only molecules with at least one ring, a molecular weight between 250 Da and 850 Da, and single bond fraction below 90%, were retained in this dataset. Ligands must have had no covalent bond to be selected. To discard ambiguities, protein chains interacting with the small molecule were characterized using their UniProtKB identifier [25].

An initial dataset was constituted by taking all the protein chains binding to at least one ligand. One protein chain per ligand was considered. If one ligand was bound to multiple protein chains, i.e., multimeric complex, then each couple chain-ligand was split and considered as a distinct unit.

Two complexes were grouped and compared to each other when both protein chains and ligands were considered as identical. In our case, two protein chains were identical when their UniProtKB was equal; ligands were considered as similar using their canonicalized SMILES fingerprint [29].

Nonetheless, having two identical proteins and ligands doesn’t necessarily represent the same binding sites, e.g., one hypothetical case involving two identical ligands bound to the two extremities of the same protein chain. Thus, molecular contacts were calculated between protein chain and ligand atoms to highlight interacting residues for every unit. The classical sum of van der Waals radii + 1.0 Å between ligand and protein atoms was used as distance threshold. To be sure, we are comparing the same binding pockets; at least 3 common interacting residues across two different units were required to make a comparison.

Thus, two similar complexes were superimposed through a structural alignment performed on common binding residues Cα. To perform such alignment, a minimum of 3 identical binding residues in the two different pockets was required. The superposition optimization followed the Kabsch algorithm [30] and was iteratively performed by favorably weighting spatially close residues at each step. 

A critical issue here is the heterogeneous atom labeling that can be a source of error in root mean square deviation (RMSD) calculation due to inverted atom labeling, particularly in ligands. To quantify structural similarity between two units, a custom root-mean square deviation (RMSD) was calculated on both ligands. Molecular fragments were generated from ligands with their corresponding center of mass calculated. Aromatic rings and functional groups were preserved while long aliphatic chains were split into multiple fragments. The Weighted fragment Root Mean Square Deviation (wRMSD_f_) similarity metric was then computed given by the formula:

(1) $${\mathrm{wRMSD}}_{f}=\sqrt{\sum_{i=1}^{N}\frac{n_{atoms\_i}}{n_{atoms\_lig}}\delta_{i}^{2}}$$

With *N* number of fragments, *n_atoms_i_* the number of atoms within the fragment *i*, *n_atoms_lig_* the total number of atoms within the molecule and *δ_i_* the distance between the fragment’s center of masses. Molecular fragmentation was performed in Pipeline Pilot R2 2017 [31].

Euclidean distances between fragments’ centers of mass were also computed. RMSD of binding residue alpha carbon was also retrieved to assess the (i) the superimposition quality, and (ii) the structural diversity in binding pockets, i.e., grasp the potential mobility of some residues in the pocket.

Using these calculated metrics, rules were defined to characterize two units as similar: (i) a wRMSD_f_ value below a threshold of 1.0 Å and (ii) no distance greater than 1.5 Å between two fragments’ center of masses. As large number of conformations can sometimes be compared between each other for highly recurrent complexes, hierarchical clustering (with complete-linkage as agglomeration method) was performed using wRMSD_f_ measure as a distance metric using R 3.4.4 [32]. 

Protein-ligand conformations were finally clustered in three categories. Units that do not share similar ligand, similar chain receptor or similar binding residues, i.e., binding sites, with other complexes, were defined as singular complexes. Cases where every conformation of one complex was identified as identical, i.e., limited structural deviation, were labeled as homogeneous complexes. Groups of conformations resulted from clustering were qualified as heterogeneous.

B-factors were retrieved from PDB structure and normalized as done in [22]. In our study, only normalized B-factors of binding residues and ligand were considered. B-factors were averaged for each residue and then averaged for each binding pockets.

## 5. Conclusions

Throughout this study, the structural diversity and redundancy of protein-ligand binding modes was assessed in the PDB for the first time. This survey of 104,777 monomeric complexes highlights the widely acknowledged redundancy in the protein-ligand context. Clustering and filtering processes have led to the description of 39,629 specific binding modes of unique protein-ligand complexes, a 2.64-fold reduction relative to the original dataset. While this type of study has been performed in the past on a smaller dataset, 2911 complexes by Drwal et al. [21], it is the first time such analysis was performed on a large scale. This research’s purpose can be used both from an analytical perspective, e.g., machine learning dataset, and applicative prospect, i.e., efficiently improving a protein-ligand complex query in a database. Such implementation will be integrated in future 3decision release.

Although the methodology offers great and innovative results compared to the approaches currently used, further improvements can be explored. For instance, RMSD of residue side chain can be computed to correlate with the observed molecular interaction. Ligand wRMSD_f_ metric can be revised by quantifying the plane rotation shifting using basic atom distance-based matching method. Finally, a layered quantification of redundancy using protein classification such as species information or PFAM classification would allow to group complexes with highly similar binding site such as kinase for instance. We also would like to explore the possibility in future developments to look specifically at allosteric compounds implicated in the investigation of catalytic, binding, or regulatory exosite [33].

## Abbreviations

ACHP_LYMST	Acetylcholine-binding protein (*Lymnaea stagnalis*)
ADP	Adenosine-5’-Diphosphate
AMP	Adenosine Monophosphate
ANP	Phosphoaminophosphonic Acid-Adenylate Ester
ATP	Adenosine-5’-Triphosphate
BACE1_HUMAN	Beta-secretase 1 (Human)
BFR_ECOLI	Bacterioferritin (*Escherichia coli*)
CAH2_HUMAN	Carbonic anhydrase 2 (Human)
CCPR_YEAST	Cytochrome c peroxidase, mitochondrial (*Saccharomyces cerevisiae*)
CDK2_HUMAN	Cyclin-dependent kinase 2 (Human)
COA	Coenzyme A
CPXA_PSEPU	Camphor 5-monooxygenase (*Pseudomonas putida*)
CPXB_BACMB	Bifunctional cytochrome P450/NADPH--P450 reductase (*Bacillus megaterium*)
CYC	Phycocyanobilin
CYC3_DESAC	Cytochrome c3 (*Desulfuromonas acetoxidans*)
DGT	2’-Deoxyguanosine-5’-Triphosphate
ESR1_HUMAN	Estrogen receptor (Human)
FAD	Flavin-Adenine Dinucleotide
FMN	Flavin Mononucleotide
GDP	Guanosine-5’-Diphosphate
GNP	Phosphoaminophosphonic Acid-Guanylate Ester
GTP	Guanosine-5’-Triphosphate
HBA_HUMAN	Hemoglobin subunit alpha (Human)
HBB_HUMAN	Hemoglobin subunit beta (Human)
HEC	Heme C
HEM	Protoporphyrin IX Containing FE
INHA_MYCTU	Enoyl-[acyl-carrier-protein] reductase [NADH] (*Mycobacterium tuberculosis*)
KAIC_SYNE7	Circadian clock protein kinase KaiC (*Synechococcus elongatus*)
NAD	Nicotinamide-Adenine-Dinucleotide
NAI	1,4-Dihydronicotinamide Adenine Dinucleotide
NAP	NADP Nicotinamide-Adenine-Dinucleotide Phosphate
NDP	NADPH Dihydro-Nicotinamide-Adenine-Dinucleotide Phosphate
NIR_THIND	Cytochrome c-552 (*Thioalkalivibrio nitratireducens*)
NOS1_RAT	Nitric oxide synthase, brain (Rat)
NOS3_BOVIN	Nitric oxide synthase, endothelial (Bos taurus (Bovine)
NQO2_HUMAN	Ribosyldihydronicotinamide dehydrogenase [quinone] (Human)
O76290_TRYBB	Pteridine reductase (*Trypanosoma brucei brucei*)
POL_HV1B1	Gag-Pol polyprotein (Human immunodeficiency virus type 1 group M subtype B)
PYRF_METH	Orotidine 5’-phosphate decarboxylase (*Methanothermobacter thermautotrophicus*)
Q8WSF8_APLCA	Soluble acetylcholine receptor (Aplysia californica)
Q9HY79_PSEAE	Bacterioferritin (*Pseudomonas aeruginosa*)
SAH	S-Adenosyl-L-Homocysteine
SAM	S-Adenosylmethionine
SAMH1_HUMAN	Deoxynucleoside triphosphate triphosphohydrolase SAMHD1 (Human)
THYX_THEMA	Flavin-dependent thymidylate synthase (*Thermotoga maritima*)
TPP	Thymidine 5′-triphosphate
UDP	Uridine-5’-Diphosphate
